# Gambogic acid and gambogenic acid induce a thiol-dependent heat shock response and disrupt the interaction between HSP90 and HSF1 or HSF2

**DOI:** 10.1007/s12192-021-01222-4

**Published:** 2021-07-30

**Authors:** Linda Pesonen, Sally Svartsjö, Viktor Bäck, Aurélie de Thonel, Valérie Mezger, Délara Sabéran-Djoneidi, Pia Roos-Mattjus

**Affiliations:** 1 Faculty of Science and Engineering, Biochemistry, Åbo Akademi University, Artillerigatan 6, 20520 Åbo/Turku, Finland; 2 Université de Paris, UMR7216 Épigénétique et Destin Cellulaire, CNRS, F-75013 Paris, France

**Keywords:** Gambogenic acid, Gambogic acid, Heat shock factor, Heat shock protein 90, Heat shock response, Thiol

## Abstract

Cancer cells rely on heat shock proteins (HSPs) for growth and survival. Especially HSP90 has multiple client proteins and plays a critical role in malignant transformation, and therefore different types of HSP90 inhibitors are being developed. The bioactive natural compound gambogic acid (GB) is a prenylated xanthone with antitumor activity, and it has been proposed to function as an HSP90 inhibitor. However, there are contradicting reports whether GB induces a heat shock response (HSR), which is cytoprotective for cancer cells and therefore a potentially problematic feature for an anticancer drug. In this study, we show that GB and a structurally related compound, called gambogenic acid (GBA), induce a robust HSR, in a thiol-dependent manner. Using heat shock factor 1 (*HSF1*) or *HSF2* knockout cells, we show that the GB or GBA-induced HSR is HSF1-dependent. Intriguingly, using closed form ATP-bound HSP90 mutants that can be co-precipitated with HSF1, a known facilitator of cancer, we show that also endogenous HSF2 co-precipitates with HSP90. GB and GBA treatment disrupt the interaction between HSP90 and HSF1 and HSP90 and HSF2. Our study implies that these compounds should be used cautiously if developed for cancer therapies, since GB and its derivative GBA are strong inducers of the HSR, in multiple cell types, by involving the dissociation of a HSP90-HSF1/HSF2 complex.

## Introduction

HSP90 is an essential ATP-dependent molecular chaperone that is one of the most abundant proteins in eukaryotic cells. HSP90 has a vast repertoire of client proteins consisting of kinases and phosphatases, nuclear hormone receptors, actin and tubulin, and the proteasome subunits, and its activity and client specificity rely on different co-chaperones (Csermely et al. 1998; Pearl 2016). There are two isoforms of cytosolic HSP90 in humans; HSP90α is considered the main isoform that is induced upon stress, whereas HSP90β is only slightly inducible and more abundant than HSP90α under physiological conditions. HSP90β is considered important for normal cellular processes such as differentiation and cytoprotection and for maintenance of the cytoskeleton (Csermely et al. 1998).

The heat shock response (HSR) is a universal stress-protective pathway that is induced in response to proteotoxic stress, e.g., exposure to heat, proteasome inhibitors, and infections (Richter et al. 2010). The HSR is characterized by a fast and massive increase in the expression of molecular chaperones, such as the heat shock proteins (HSPs), which refold damaged proteins and prevent protein aggregation. The transcription of *HSP* genes is mediated by heat shock factors (HSFs). Upon stress, HSFs oligomerize and accumulate into the nucleus and bind to specific heat shock elements (HSEs). During heat stress, hundreds of genes are upregulated, and thousands are downregulated (Mahat et al. 2016; Vihervaara et al. 2017). HSF1 is regarded as the master regulator of the HSR in mammals, whereas HSF2 is involved in differentiation and development (Joutsen and Sistonen 2019). HSF2 has, however, been shown to form heterocomplexes with HSF1 and modulate the expression of HSR genes, suggesting also a role in the HSR (Östling et al. 2007; Sandqvist et al. 2009).

According to the chaperone titration model, cytoplasmic HSF1 monomers are kept inert when complexed with chaperones, such as HSP70 and HSP90 (Gomez-Pastor et al. 2018). Today, it is still unclear whether HSF2 can also form complexes with chaperones. Upon protein-damaging stress, chaperones are required for folding of denatured proteins, and HSF1 is released from the chaperone complex, trimerized and activated. The mechanism by which HSF1 is inactivated is not completely clear. HSF1 has been shown to be inactivated by distinct post-translational modifications and by negative feedback regulation by HSPs, in particular HSP70 and HSP40 (Kmiecik et al. 2020; Masser et al. 2020). Interestingly, HSP90 was also recently shown to bind to HSF1 trimers and to favor the release of HSF1 from HSEs (Kijima et al. 2018). Clearly, the interaction between HSF1 and HSP90 is multifaceted.

Due to the notion that many HSP90 clients have crucial roles in rapidly growing cancer cells, inhibition of HSP90 suppresses many signaling pathways that are important for cancer progression. A number of HSP90 inhibitors have been extensively studied, but none of these compounds have been approved for clinical use by the FDA (Yuno et al. 2018). The first isolated HSP90 inhibitors were geldanamycin and radicicol (Neckers and Workman 2012). These compounds were shown to bind to the N-terminal ATP-binding pocket of HSP90 by mimicking the conformation of ATP. However, although inhibiting HSP90 activity, these proved too toxic, insoluble, and metabolically unstable for clinical use (Neckers and Workman 2012). Several compounds have been synthesized using geldanamycin and radicicol as templates, including 17-AAG (17-allylamino-17-demethoxygeldanamycin, tanespimycin), 17-DMAG, STA-9090, and AUY-922. These N-terminal HSP90 inhibitors, also called classic inhibitors, are the most studied inhibitors. However, most of them induce a HSR that stimulates the synthesis of HSP90, which is counterproductive when considering treating cancer (Neckers and Workman 2012; Yuno et al. 2018).

Many bioactive natural compounds found in plants have been shown to inhibit HSP90. Celastrol (tripterine), a pentacyclic triterpenoid derived from the plant thunder god vine (*Tripterygium wilfordii*), is an anti-inflammatory agent that has long been used in Chinese medicine to treat autoimmune and inflammatory diseases (Chen et al. 2018). Celastrol has been shown to inhibit angiogenesis, migration, and invasion and to suppress cancer progression, by affecting multiple targets in cancer cells, including the IKK-NF-κB pathway, HSP90, and the proteasome (Chen et al. 2018). Gambogic acid (GB) is a natural product derived from the gamboge resin of the *Garcinia hanburyi* tree, which similarly to celastrol has also been used in Chinese medicine (Banik et al. 2018). GB is a polyprenylated xanthone with antitumor, antimicrobial, and anti-inflammatory effects (Banik et al. 2018; Kashyap et al. 2016). GB also suppresses the progression of many cancers in vitro and in vivo (Tang et al. 2017; Wu et al. 2004; Xia et al. 2017), and in China, it has been evaluated in a clinical trial focused on targeting advanced malignant cancers (Chi et al. 2013). GB, as celastrol, affects different target proteins in the cell, such as HSP90, NF-κB, c-Myc, PI3K, p-AKT, MDM2, and the proteasome (Banik et al. 2018; Kashyap et al. 2016). Gambogenic acid (GBA) is another active ingredient of the resin of *Garcinia hanburyi* (Asano et al. 1996). GBA resembles GB but has a geranyl and a hydroxyl group instead of the ether ring in GB (Asano et al. 1996). GBA, like GB, is toxic to many different cancer cell lines (Huang et al. 2019; Liu et al. 2017; Zhou et al. 2013). Whether GBA induces a HSR has not been studied before.

In this study, we show that acute treatment with GB and GBA induces a robust HSR in multiple cell types irrespective of their developmental origins. Furthermore, we establish that GB and GBA induce the HSR in a thiol-dependent manner. In addition, we show that GB or GBA treatment disrupt the protein-protein interaction between HSP90 and HSF1 and/or between HSP90 and HSF2. The potential of GB and GBA to activate the HSF pathway could be detrimental in cancer therapies and should be carefully considered if using GB or GBA in treatments or for further drug development.

## Materials and methods

### Generation of *HSF1* knockout U2OS cells with CRISPR-Cas9

The human osteosarcoma U2OS cells (HTB-96, ATCC) *HSF1* knockout cells (*HSF1* KO) were generated with CRISPR-Cas9 as previously described for *HSF2* knockout U2OS cells (*HSF2* KO) (Joutsen et al. 2020). *HSF1* KO cell clones were genotyped by DNA sequencing of PCR products spanning the targeted region of the *HSF1* gene. The selected U2OS clone presented one single base insertion on *HSF1* exon 3 (Table 1), and the sequence analysis of six independent PCR products shows the same mutation, suggesting that all the alleles have the same mutation. Guide RNA sequence targeting *HSF1* exon 3: 5’-TGTTATGTGCAGATGGCTTC-3’. The following primers were used for PCR for validation: forward (hHSF1_Cr_ex3_F): 5′-GGTCCTTGTGGGTATGAACCT-3′ and reverse (hHSF1_Cr_ex3_R): 5′-CACACTGGTCACTTTCCTCTTG-3′.

**Table 1:** CRISPR-Cas9-induced mutation in HSF1 allele

	**Sequence**	**Mutation**	**Protein product**
WT	**GGTCCTTGTGGGTATGAACCT**GGGGTCCCCATGGAAGAACCGTGAAGCCGGAGCTGTACTCCACGTGTGTCGGGCGCAGGGAGCCCTGTGGGGACACAGGGTCTCCCTTAGACCAAGGCCACTCGGCCACCCAGGCATGGGCTCTGAGGGGGCAGGGCAGGGTCTGACCATGGCCAAGCCCCGCAGCAGCCTCCTGGAGCAGTGGCCGCTCTTCAGGGGTTCTGGTCCCGCCCTGAGGCAGAGCTGCCCCCTTCCCTGTTATGTGCAGATGGCTTCCGGAAAGTGGTCCACATCGAGCAGGGCGGCCTGGTCAAGCCAGAGAGAGACGACACGGAGTTCCAGCACCCATGCTTCCTGCGTGGCCAGGAGCAGCTCCTTGAGAACAT**CAAGAGGAAAGTGACCAGTGTG**		WT HSF1 protein
All alleles sequenced “*HSF1* KO”	**GGTCCTTGTGGGTATGAACCT**GGGGTCCCCATGGAAGAACCGTGAAGCCGGAGCTGTACTCCACGTGTGTCGGGCGCAGGGAGCCCTGTGGGGACACAGGGTCTCCCTTAGACCAAGGCCACTCGGCCACCCAGGCATGGGCTCTGAGGGGGCAGGGCAGGGTCTGACCATGGCCAAGCCCCGCAGCAGCCTCCTGGAGCAGTGGCCGCTCTTCAGGGGTTCTGGTCCCGCCCTGAGGCAGAGCTGCCCCCTTCCCTGTTATGTGCAGATGG**C**CTTCCGGAAAGTGGTCCACATCGAGCAGGGCGGCCTGGTCAAGCCAGAGAGAGACGACACGGAGTTCCAGCACCCATGCTTCCTGCGTGGCCAGGAGCAGCTCCT***TGA***GAACAT**CAAGAGGAAAGTGACCAGTGTG**	1 nt insertion (C)	Putative 112 amino-acid truncated protein (with only the first 76 aa identical to HSF1), but is never detected

In bold: PCR primer position. Underlined: exon3. Double underlined: stop codon.

### Cell culture and experimental treatments

HeLa (human cervical cancer, CCL-2, ATCC), HEK293 (human embryonic kidney cells, CRL-1573, ATCC), HDF (primary human dermal fibroblasts, PCS-201-010, ATCC), and U2OS (WT (HTB-96, ATCC), *HSF1* KO, and *HSF2* KO) cells were cultured in the same conditions: they were maintained at 37 °C in a humidified 5% CO_2_ atmosphere and cultured in Dulbecco’s modified Eagle’s medium (D6171, Sigma-Aldrich) supplemented with 10% fetal calf serum, 2 mM L-glutamine, and 100 U/ml penicillin-100 μg/ml streptomycin mixture. RWPE-1 (human normal prostate epithelial cells, CRL-11609, ATCC) cells were cultured in Keratinocyte Serum Free Medium (17005042, Gibco) supplemented with 0.05 mg/ml bovine pituitary extract, 5 ng/ml epidermal growth factor, and 100 U/ml penicillin-100 μg/ml streptomycin mixture.

To induce a HSR and/or inhibit HSP90, cells were treated with either 17-AAG (17A, ant-agl-5, InvivoGen), celastrol (Cel, 3203, Tocris Bioscience), gambogic acid (GB, 3590, Tocris Bioscience), or gambogenic acid (GBA, BP2014, Chengdu Biopurify Phytochemicals Ltd.). The inhibitors were diluted in DMSO before treating the cells with concentrations indicated in the figures. Control cells were treated with DMSO only. Heat shock treatments were conducted on cell dishes wrapped in Parafilm and submerged in a water bath at 42 °C for indicated times. For the recovery phase, after treatments, the cells were placed in an incubator at 37 °C for 3 h after removing the parafilm. In order to investigate the thiol reactiveness of GB and GBA, cells were, in addition to GB and GBA, also treated with dithiothreitol (DTT), GB + DTT and GBA + DTT, respectively. DTT was added in a 10-fold excess to GB and GBA and left to react for 15 min at room temperature prior to cell treatment, with the indicated concentrations and the times, as described in the figure legends.

### Immunoblot analysis

Cells were lysed with either lysis buffer (25 mM HEPES pH 8.0, 100 mM NaCl, 5 mM EDTA pH 8.0, 0.5% Triton X-100) or Laemmli sample buffer (30% glycerol, 3% SDS, 187.5 mM Tris-HCl pH 6.8, 0.015% bromophenol blue, 3% β-mercaptoethanol). Cells lysed with lysis buffer were incubated in the buffer containing protease and phosphatase inhibitor cocktails (04693159001 and 04906845001, Roche), 1 mM serine protease inhibitor PMSF (phenylmethylsulfonyl fluoride), and 0.5 mM DTT for 10 min at 4 °C and centrifuged at 16400 rpm (25 000 g) for 10 min at 4 °C. Cells lysed with Laemmli buffer were suspended in an appropriate amount of 3 × Laemmli buffer and boiled for 5–10 min. The protein concentration of the lysates in lysis buffer was determined using the Bradford method.

Cell lysates were resolved on an 8% sodium dodecyl sulfate-polyacrylamide gel (SDS-PAGE) and transferred to a 0.45-μm pore size nitrocellulose membrane (Protran). The membranes were boiled for 10 min in ultrapure water H_2_O directly after transfer and blocked with 5% skimmed milk powder in 0.3% PBS-Tween20. The primary antibodies were diluted in PBS containing BSA and 0.02% NaN_3_. The membranes were incubated with the primary antibodies overnight at 4 °C. The primary antibodies used were anti-HSF1 (ADI-SPA-901, Enzo Life Sciences), anti-HSF2 (clone 3E2, MAB88079, Sigma-Aldrich or HPA031455, Sigma-Aldrich), anti-HSP70 (ADI-SPA-810, Enzo Life Sciences), anti-FLAG (clone M2, F3165 M2ab, Sigma-Aldrich), anti-Myc (clone 9E10, M5546, Sigma-Aldrich), and anti-β-tubulin (clone AA2, T8328, Sigma-Aldrich). Secondary antibodies conjugated to horseradish peroxidase were purchased from Promega, GE Healthcare, or Abcam. The amount of relative HSP70 protein related to β-tubulin was quantified with ImageJ (n=2–5).

### Biotin-mediated oligonucleotide pulldown assay

The biotin-mediated oligonucleotide pulldown assay is modified from Anckar et al. (2006). U2OS WT cells were lysed with buffer C (25% glycerol, 0.42 M NaCl, 1.5 mM MgCl_2_, 0.2 mM EDTA, pH 8.0, 20 mM HEPES) containing 0.5 mM DTT and 0.5–1 mM PMSF. Buffer C extracts (150–200 μg protein) were incubated in binding buffer (20 mM Tris-HCl, pH 7.5, 100 mM NaCl, 2 mM EDTA, 10% glycerol) with 3 μg annealed oligonucleotides, containing either a heat shock element (HSE) or a scrambled sequence and 0.5 μg/μl salmon sperm DNA (Sigma-Aldrich). The HSE-containing oligonucleotides 5’-biotin-TCGACTAGAAGCTTCTAGAAGCTTCTAG-3’ and 5’-CTAGAAGCTTCTAGAAGCTTCTAGTCGA-3’ (Vuori et al. 2009) and the scrambled control oligonucleotides 5’-biotin-AACGACGGTCGCTCCGCCTGGCT-3’ and 5’-AGCCAGGCGGAGCGACCGTCGTT-3’ (Anckar et al. 2006) were purchased from Oligomer. The proteins were allowed to bind to the oligonucleotides for 20 min at room temperature and 30 min at 4 °C. The samples were precleared with Glutathione Sepharose 4 Fast Flow (17-5132-01, GE Healthcare) for 30 min at 4 °C under rotation. The remaining DNA was precipitated with 25 μl Streptavidin-Sepharose 4B (434341, Invitrogen) for 1 h at 4 °C under rotation. Bound fractions were washed three times with binding buffer and twice with binding buffer containing 0.2% Triton X-100. The DNA-bound proteins were suspended in 20 μl 3× Laemmli buffer and boiled for 5 min to elute the proteins. The samples were analyzed with SDS-PAGE and immunoblotting.

### Quantitative real-time reverse transcription-PCR

RNA was isolated from U2OS and HeLa cell pellets using a Nucleospin RNA isolation kit (Macherey-Nagel) according to the manufacturer’s instructions, and the RNA concentration was measured using a NanoDrop 2000 spectrophotometer (Thermo Fisher Scientific). The reverse transcriptase enzyme M-MLV RT (H-) (Moloney Murine Leukemia Virus Reverse Transcriptase RNase H minus, Promega) was then used to transcribe 1 μg of total RNA to cDNA using Oligo(dT)_15_ primers (Promega). KAPA Probe Fast qPCR Master Mix (2X) ABI Prism (for *HSPA1A* (*HSP70)*, *HSPH1* (*HSP110)*, and *18S* RNA) or a KAPA SYBR FAST qPCR Master Mix (2X) ABI Prism (for *satIII* and *hGAPDH*) kits (KK4706 and KK4604, KapaBiosystems) were used for the qRT-PCR reactions, and these were performed with a StepOnePlus Real-Time PCR system (Applied Biosystems). Primers and probes were purchased from Oligomer and Roche Universal Probe Library and can be found in Table 2. The relative quantities of *HSPA1A* and *HSPH1* were normalized against *18S* rRNA with the help of a standard curve. The relative quantities of *satIII* were normalized against *hGAPDH*. The fold induction was calculated against the respective mRNA levels in control cells. All reactions were run in triplicate from samples, generally derived from at least three biological replicates.

**Table 2:** Primers (*F* forward, *R* reverse) and probes used for the qPCR reactions

**Gene**	**Primer**	**Probe**	**Publication**
*hHSPA1A/hHSP70.1*	F 5′-GCCGAGAAGGACGAGTTTGA-3′ R 5′-CCTGGTACAGTCCGCTGATGA-3′	5′-Fam-TTACACACCTGCTCC AGCTCCTCCCTCTT-BHQ1-3′	Östling et al. (2007)
*hHSPH1/hHSP110*	F 5′-AGCCATGTTGTTGACTAAGCTG-3′ R 5′-TCTGTAAAGAAGGAGGGGACTG-3′	#90	Vihervaara et al. (2013)
*18S* rRNA	F 5′-GCAATTATTCCCCATGAACG-3′ R 5′-GGGACTTAATCAACGCAAGC-3′	5′-Fam-TCCCAAGTAAGTGCGGGTC-BHQ1-3′	Budzynski et al. (2015)
*SatIII*	F 5′-AATGGAATGCAATGGAATGG-3′ R 5′-CCTGTACTCGGGTTGATTCC-3′	SYBR Green	Sandqvist et al. (2009)
*hGAPDH*	F 5-ACCCACTCCTCCACCTTTGA-3′ R 5-TTGCTGTAGCCAAATTCGTTGT-3′	SYBR Green	

Probe #90 (04689151001) is from the Universal ProbeLibrary (Roche Applied Science).

### Statistical analysis

Statistical analyses were performed using GraphPad Prism 7 Software (GraphPad Prism Software, http://www.graphpad.com). The data was analyzed using one-way ANOVA and corrected with the Holm-Šídák’s multiple comparisons test. The significance level was set to 0.05. Mean + SEM is shown in the figures.

### Immunofluorescence

To detect nuclear stress bodies, immunofluorescence was performed as in Sandqvist et al. (2009). Briefly, HeLa cells were cultured on coverslips and fixed with 100% methanol for 6 min at 4 °C. The methanol was aspirated, and the cells were washed three times with 0.05% PBS-Tween20. The cells were incubated in a blocking solution containing 10% BSA (bovine serum albumin) in 0.05% PBS-Tween20 for 1 h. The cells were incubated with anti-HSF1 antibody (Holmberg et al. 2000) diluted in blocking solution (1:300) overnight at 4 °C. The secondary antibody (anti-rabbit IgG, Alexa Fluor 488) was diluted in blocking solution (1:700) and added for 1 h. The coverslips were mounted using VECTASHIELD mounting medium (Vector Laboratories), and the cells were visualized with an LSM510-Meta scanning confocal microscope (Carl Zeiss).

### HSP90-HSF1 interaction assay

HSP90-HSF1 interaction was studied as in Kijima et al. (2018). HEK293 cells were transfected with WT HSF1 and either of the two HSP90 mutants: FLAG-HSP90α E47A and FLAG-HSP90β E42A. Both mutant constructs were a kind gift from Dr. Len Neckers (NIH, Bethesda, USA). These mutants are in a closed conformation and are described in detail in Kijima et al. (2018). Transfections were performed using the Neon Transfection System (Thermo Fisher Scientific) according to the manufacturer’s instructions. 7 × 10^6^ HEK293 cells were suspended in 100 μl Resuspension Buffer R and mixed with plasmids for Myc-His-HSF1 WT (described in Westerheide et al. (2009)), pBlueScript empty vector (BS), FLAG-HSP90α E47A, or FLAG-HSP90β E42A. The BS vector was used as a negative control. The cells were subjected to electroporation (1245 V, 10 ms pulse width, 3 pulses), plated, and left to recover in culture medium for 48 h before treatments.

For immunoprecipitation, the cells were lysed with TGNET buffer (50 mM Tris HCl pH 7.5, 5% Glycerol, 100 mM NaCl, 2 mM EDTA, 0.5% Triton X-100) containing 1 mM PMSF and protease and phosphatase inhibitor cocktails (04693159001 and 04906845001, Roche). Lysates were incubated on ice for 10 min and centrifuged at 16400 rpm (25 000 g) for 15 min at 4 °C. The protein concentrations of the supernatants were determined using the Bradford method, and 15 μg of protein was subjected to SDS-PAGE for protein expression analyses. Seven hundred micrograms of protein was subjected to immunoprecipitation using 30 μl of anti-FLAG M2 affinity beads (A2220, Sigma-Aldrich). The beads were incubated with rotation for 2 h, at 4 °C, and then centrifuged at 6000 rpm (3500 g) for 30 s. The beads were washed three times with TGNET buffer and then suspended in 12 μl 3 × Laemmli buffer and boiled for 5 min to elute the proteins. The samples were analyzed with SDS-PAGE and immunoblotting.

## Results

### Gambogic acid induces a heat shock response in multiple cell lines

**Fig. 1:**
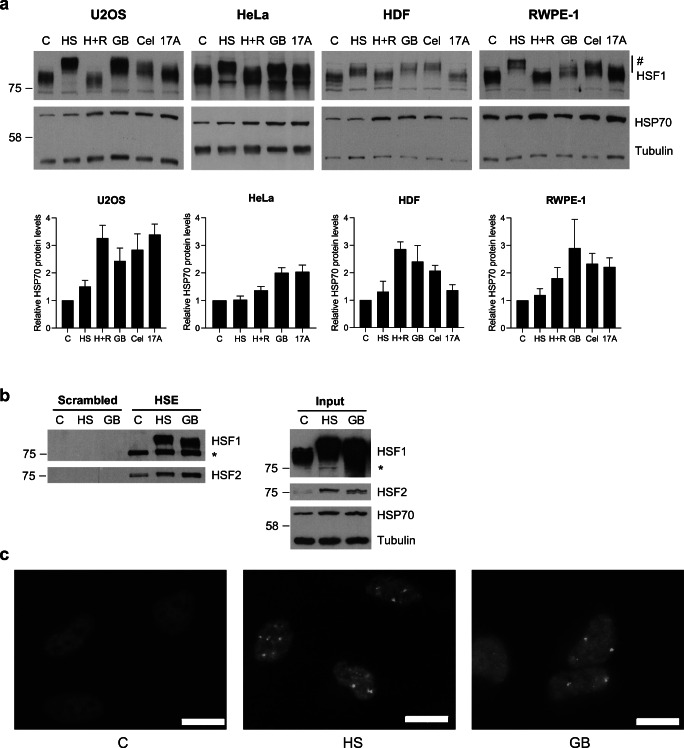
Acute treatment with gambogic acid induces a heat shock response (HSR). **a** Immunoblot analysis of HSF1 and HSP70 expression. U2OS, HeLa, HDF, and RWPE-1 cells were treated with heat shock (HS, 42 °C, 1 h), 1 h HS with 3-h recovery at 37 °C (H+R), and gambogic acid (GB), celastrol (Cel) or 17-AAG (17A) for 4 h. U2OS: 1.25 μM GB and Cel, 0.25 μM 17A; HeLa: 2 μM GB, 1.5 μM 17A; RWPE-1: 1.25 μM GB and Cel, 3 μM 17A; HDF: 2.5 μM GB and Cel, 3 μM 17A. The HSF1 uppershift induced by HS and treatments (labeled with #) correspond to hyperphosphorylated HSF1 forms. β-tubulin was used as a loading control. The amount of relative HSP70 protein related to β-tubulin was quantified with ImageJ (*n*=2-4). **b** Oligonucleotide-mediated pulldown of HSF1 and HSF2 in WT U2OS cells, untreated (C), treated with heat shock 42 °C for 1 h (HS) or with 1.25 μM gambogic acid for 4.5 h (GB). Input indicates total cell lysates from treated cells. β-tubulin serves as a loading control. Single asterisk indicates previously blotted HSF2. **c** Immunofluorescence staining of endogenous HSF1 in nuclear stress bodies in HeLa cells. GB: 4 μM GB, HS: 2 h heat shock (HS, 42 °C) (n=3). Scale bar: 20 μm. Mean ± SEM shown

GB has been extensively studied in the context of cancer and shown to work as an anticancer agent (Banik et al. 2018). However, there are contradicting reports whether GB induces a HSR (Davenport et al. 2011; Yim et al. 2016), which is cytoprotective for cancer cells and therefore a potentially problematic feature for an anticancer drug. Therefore, to determine if GB indeed induces a HSR, we treated both transformed (U2OS and HeLa) and untransformed (HDF and RWPE-1) cells with acute treatments of GB (Fig. 1a). HSF1 hyperphosphorylation and upregulation of HSP70 protein levels were used as proxies for the activation of the HSR (Sarge et al. 1993). We included the previously defined HSP90 inhibitors celastrol and 17-AAG (17-allyllaminogeldanamycin) in the analyses, since both are known activators of HSF1 and subsequent HSR (Bagatell et al. 2000; Westerheide et al. 2004). As a positive control for HSF1 hyperphosphorylation, we used 1-h heat shock (HS) treatment at 42 °C, and for HSP70 protein upregulation, HS + 3 h recovery (H+R) was used. In both untransformed and transformed cell lines, acute treatments with GB induced HSF1 hyperphosphorylation and increased HSP70 levels to a similar extent as HS (Fig. 1a). Moreover, HSF1 hyperphosphorylation and HSP70 upregulation were also induced by celastrol in all cell lines, whereas 17-AAG at these concentrations induced an HSR in all cell lines except primary HDFs. Altogether, these results demonstrate that GB induces a robust HSR in untransformed and transformed human cells.

To further address whether GB treatment results in increased HSF activity, we employed an oligopulldown assay to study both HSF1 and HSF2 DNA-binding capacity (Fig. 1b). Lysates from treated cells were subjected to a biotinylated heat shock element (HSE)-containing oligo or a scrambled oligo, which were purified with streptavidin beads together with their respective binding proteins. HSF2 bound to the HSE-oligo already in untreated cells, and the binding was increased after treatments with GB or HS (Fig. 1b), in agreement with previous results in heat-shocked cells using chromatin immunoprecipitation (Ahlskog et al. 2010). In contrast, HSF1 DNA binding was detected only after HS or GB treatment. Therefore, acute treatment with GB impacts the DNA-binding activities of both HSF1 and HSF2, in a manner similar to heat shock.

HSF1 and HSF2 localize to subnuclear structures called nuclear stress bodies (nSBs) upon heat stress (Alastalo et al. 2003; Jolly et al. 2002). These nSBs form on areas with pericentromeric heterochromatin, where HSF1 induces transcription of noncoding *satellite III* (*satIII*) RNA. The function of the noncoding RNAs is not well understood, but the transcripts have been suggested to affect chromatin organization and recruitment of transcription and splicing factors (Biamonti and Vourc'h 2010), and HSF1 recruitment to the nSBs is a hallmark of the HSR in human cells. Using indirect immunofluorescence, probing for endogenous HSF1, we studied the localization of HSF1 after HS and GB treatment. We demonstrated that GB induces HSF1 localization to nSBs in HeLa cells, similarly to HS (Fig. 1c). Taken together, our results show that the HSR induced by acute treatment with GB activates both HSF1 and HSF2 in multiple human cell lines of different origin.

### GB and GBA induce a heat shock response in a thiol-dependent manner

**Fig. 2:**
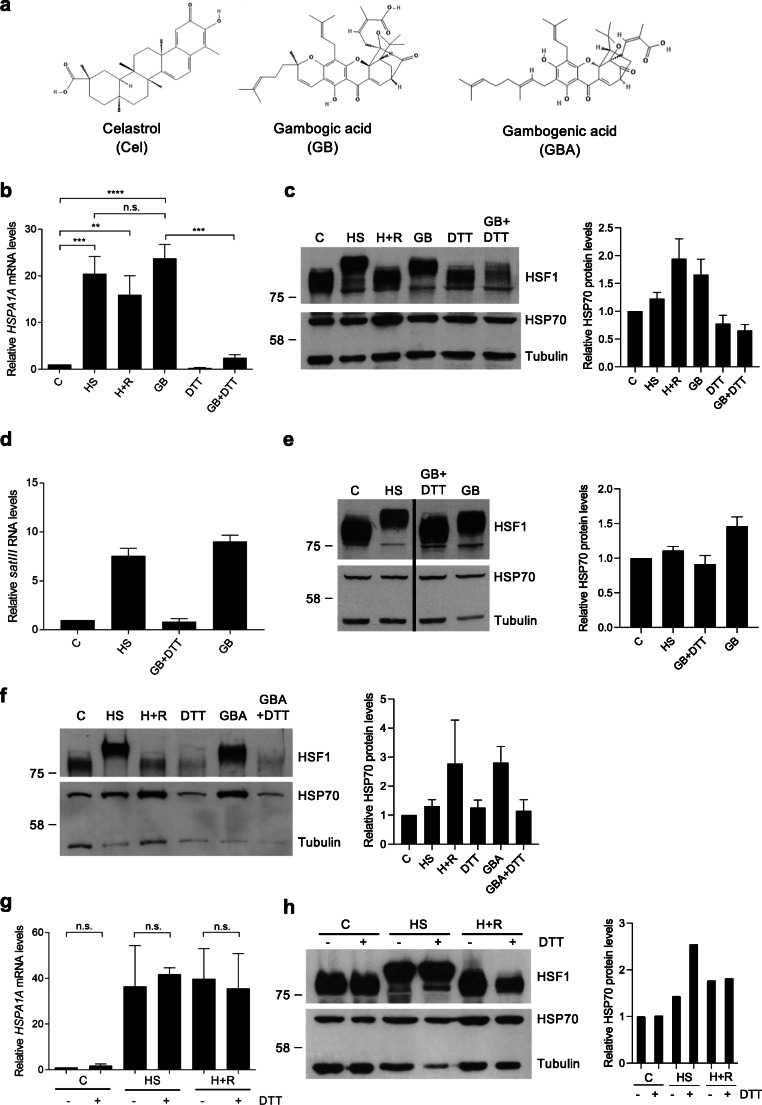
Gambogic acid and gambogenic acid induce a HSR in a thiol-dependent manner. **a** Molecular structures of celastrol, gambogic acid (GB), and gambogenic acid (GBA). The structures are taken from PubChem (https://pubchem.ncbi.nlm.nih.gov, PubChem IDs: 122724, 15559465, and 10794070) and modified. **b** qRT-PCR of *HSPA1A (HSP70)* mRNA of WT U2OS cells treated with 1 h heat shock at 42 °C (HS) or with 1 h HS followed by 3 h recovery at 37 °C (H+R), 1.25 μM GB for 4.5 h or with GB pretreated with 10×DTT for 15 min prior to adding the mixture to the cells (GB+DTT) or with 12.5 μM DTT alone for 4.5 h. *HSPA1A (HSP70)* mRNA normalized to 18S rRNA (*n*=3). One-way ANOVA, mean ± SEM shown. ** p≤ 0.01, *** p≤0.001, ****p≤0.0001, n.s. p >0.05. **c** Immunoblot analysis of HSF1 and HSP70 of the corresponding samples in **b**. β-tubulin was used as a loading control. **d** qRT-PCR of *satIII* transcripts in HeLa cells treated with 1 h HS (42 °C) or for 4 h with 4 μM GB or 4 μM GB pretreated with 10×DTT (GB+DTT). qRT-PCR with SYBR Green, normalized to *hGAPDH* (*n*=2). Mean ± SEM shown. **e** Immunoblot analysis of HSF1 and HSP70 expression of the corresponding samples in **d**. β-tubulin was used as a loading control. **f** Immunoblot analysis of U2OS cells treated with 1 h heat shock at 42 °C (HS) or with 1 h HS followed by 3-h recovery at 37 °C (H+R), 2.5 μM GBA for 4.5 h or with GBA pretreated with 10×DTT for 15 min prior to adding the mixture to the cells (GB+DTT) or with 25 μM DTT alone for 4.5 h. **g** qRT-PCR of *HSPA1A* mRNA from WT U2OS cells that were treated with heat shock (HS, 42 °C, 1 h), 1 h HS with 3 h recovery at 37 °C (H+R), with or without 12.5 μM DTT (−/+DTT) prior to treatment. C+DTT samples were treated with 12.5 μM DTT for 1 h. *HSPA1A* mRNA normalized to *18S* rRNA (*n*=3). One-way ANOVA, mean ± SEM shown. n.s. p >0.05. **h** Immunoblot analysis of HSF1, HSF2, and HSP70 of the corresponding samples in **g**. β-tubulin was used as a loading control. The amount of relative HSP70 protein related to β-tubulin was quantified with ImageJ (*n*=1–4) for the western blots. Mean ± SEM shown

The natural products, celastrol and GB, have similar chemical features, as they both contain an α,β -unsaturated ketone moiety (Fig. 2a). The resin from *Garcinia hanburyi* contains an additional active compound called gambogenic acid (GBA; Fig. 2a), which also contains an α,β-unsaturated ketone moiety. The biological effects of celastrol can be inhibited by the excess of free thiol, suggesting that celastrol reacts with key thiols in proteins (Lee et al. 2006; Peng et al. 2010; Trott et al. 2008). To examine whether GB is also thiol-responsive and inactivated by the excess of free thiols, we incubated GB with 10-fold excess dithiothreitol (DTT) before applying it to the cells. Intriguingly, the results showed that GB’s ability to induce *HSP* gene expression was inactivated by DTT, as evidenced by significantly lower *HSPA1A* (*HSP70*) mRNA expression levels in cells treated with a GB+DTT mixture (Fig. 2b). Moreover, extensive HSF1 hyperphosphorylation was also abolished after GB+DTT treatment (Fig. 2c). We also determined the amount of *satIII* transcripts produced after treatment with GB. In agreement with nSB formation (Fig. 1c), we observed that the transcription of *satIII* RNA was induced by GB, as well as HS (Fig. 2d). The GB-induced *satIII* transcripts were not produced if GB was pretreated with DTT before addition to the cells (Fig. 2d, e), demonstrating that GB can indeed be inhibited by the excess of thiols. We also addressed whether GBA can elicit a HSR. We found that acute GBA treatments also induced a HSR and that GBA was inactivated by incubation with excess DTT, suggesting that both GB and GBA act in a thiol-dependent manner on the triggering of a HSR (Fig. 2f).

Huang et al. (1994) showed that the HSR can be inhibited by thiol-reducing agents (e.g., 2 mM DTT) (Huang et al. 1994). To rule out that DTT does not, by itself, inhibit the HSR in our experiments, we treated cells before HS treatment with the same concentrations of DTT as in Fig. 2 b and c (12.5 μM) and assessed *HSPA1A (HSP70)* mRNA and protein levels, as well as HSF1 hyperphosphorylation. We observed normal HSR and recovery profiles, showing that 12.5 μM DTT does not inactivate the HSR by itself (Fig. 2g, h). Therefore, we conclude that GB and GBA, as celastrol, are thiol-responsive chemicals and that pretreatment with DTT inactivates GB and GBA and therefore perturbs the GB/GBA-induced activation of the HSR.

### The GB- or GBA-induced HSR is HSF1-dependent

**Fig. 3:**
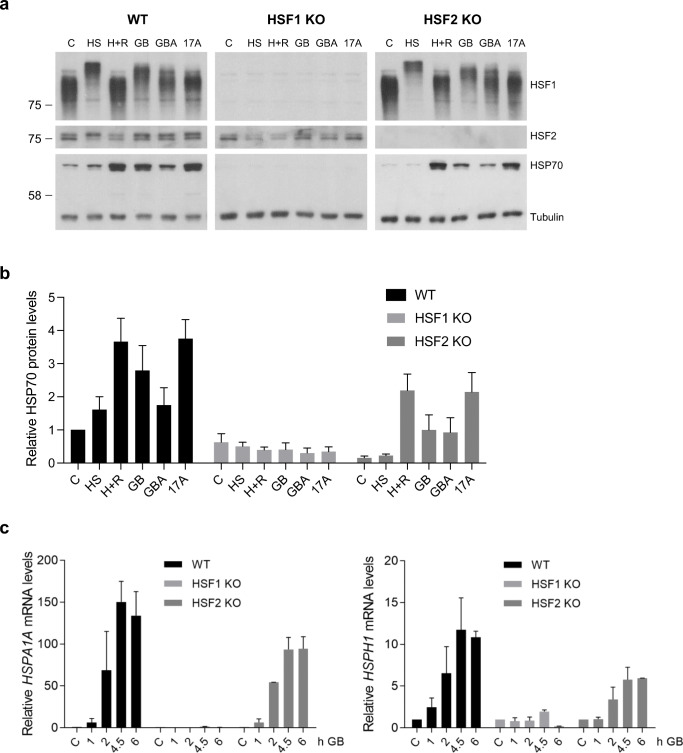
Gambogic acid and gambogenic acid induce a heat shock response (HSR) in an HSF1-dependent manner, whereas HSF2 is dispensable for the HSR. **a** WT, *HSF1*, and *HSF2* knockout (KO) U2OS cells were treated with heat shock (HS, 42 °C, 1 h), 1 h HS with 3-h recovery (H+R), 1.25 μM gambogic acid (GB), 2.5 μM gambogenic acid (GBA), or 0.5 μM 17-AAG for 4.5 h. Immunoblot of HSF1, HSF2, and HSP70. β-tubulin serves as a loading control. **b** The amount of relative HSP70 protein related to β-tubulin was quantified with ImageJ (*n*=3), **c** WT, *HSF1* KO, and *HSF2* KO U2OS cells were treated with 1.25 μM GB for indicated times. qRT-PCR of *HSPA1A (HSP70)* and *HSPH1 (HSP110)* mRNA, normalized to *18S* rRNA (*n*=2). Mean ± SEM shown

To study whether HSF1 and HSF2 are required for GB- or GBA-induced HSR, we treated WT U2OS cells as well as CRISPR-generated *HSF1* or *HSF2* knockout (KO) U2OS cells with GB and GBA. 17-AAG was used as positive control, because it has been shown to induce a HSF1-dependent HSR (Bagatell et al. 2000). Using immunoblotting, we show that all compounds induced a robust HSF1 hyperphosphorylation and upregulation in HSP70 protein expression levels in WT cells (Fig. 3a, b). No induction of HSP70 protein was detected with any of the treatments in *HSF1* KO cells (Fig. 3a, b). In cells lacking HSF2, HSF1 was robustly hyperphosphorylated in response to GB and GBA, but less in response to 17-AAG. There seems to be slightly less HSP70 protein in cells lacking HSF2.

The effect of GB on mRNA levels of *HSPA1A* (*HSP70*) and *HSPH1* (*HSP110*) at different time points in WT, *HSF1* KO, and *HSF2* KO U2OS cells was investigated using quantitative RT-PCR. As demonstrated in Fig. 3c, both *HSPA1A* and *HSPH1* mRNA levels increased time-dependently after treatment with GB both in WT and in *HSF2* KO cells, albeit the levels of *HSPA1A* and *HSPH1* mRNA were lower in *HSF2* KO cells. There was no induction of *HSPA1A* or *HSPH1* mRNA in response to GB treatment in cells lacking HSF1.

These results show that 17-AAG, GB, and the GB-analog GBA induce a HSR that is strictly dependent on HSF1 but not on HSF2. In accordance with previous studies on HS-induced HSR (Joutsen et al. 2020; Östling et al. 2007; Vihervaara et al. 2013), HSF2 indeed modulates the HSR induced by GB and the other compounds.

### GB and GBA disrupt the interaction between HSP90 and HSF1 or HSF2

**Fig. 4:**
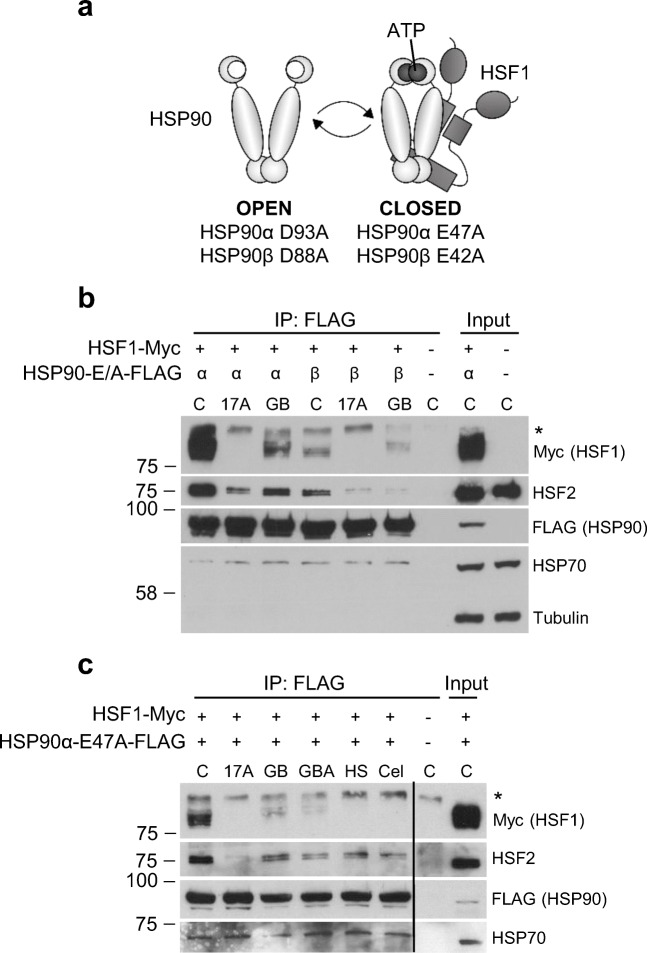
Gambogic acid and gambogenic acid induce a heat shock response by disrupting HSP90-HSF1/HSF2 interaction. **a** Schematic model of the HSP90 mutants. The closed form mutants can interact with HSF1, whereas the open mutants cannot (Kijima et al. 2018). **b** HEK293 cells were transfected with either FLAG-HSP90α E47A (α) or FLAG-HSP90β E42A (β) (closed form mutants) and HSF1-Myc and treated 2 days later with 10 μM 17-AAG (17A) or 1.5 μM gambogic acid (GB) for 4 h. BlueScript empty vector (-) was transfected as a negative control. HSP90-FLAG was precipitated with FLAG-beads, and exogenous HSF1 (Myc), HSF2, exogenous HSP90 (FLAG), and HSP70 were analyzed by immunoblotting. β-tubulin was used as a loading control in the whole cell lysates (WCL). Single asterisk indicates an unspecific band. **c** HEK293 cells were transfected with FLAG-HSP90α E47A and HSF1-Myc and treated 2 days later for 4h with 10 μM 17-AAG (17A), 1.6 μM gambogic acid (GB), 3.2 μM gambogenic acid (GBA), 3 μM celastrol (Cel), or heat shocked (3 h, 42 °C). BlueScript empty vector (-) was transfected as a negative control. The samples were analyzed as in **b**

GB has been proposed to be an HSP90 inhibitor (Davenport et al. 2011; Yim et al. 2016). The proposed model of action for HSP90 inhibitors inducing a HSR is that they disrupt HSP90-HSF1 interaction, thereby freeing HSF1 that can be activated (Gomez-Pastor et al. 2018). However, the HSP90-HSF1 interaction is transient and weak (Zou et al. 1998), and it is challenging to co-immunoprecipitate HSF1 with HSP90 without crosslinking. HSP90 functions as a dimer and cycles between a closed ATP-bound state and an open state where ATP is hydrolyzed or absent (Pearl 2016). By mutating glutamic acid residues to alanines at positions 47 in HSP90α and 42 in HSP90β (E47A and E42A, respectively), Kijima et al. (2018) generated two HSP90 mutants that are constantly in the closed ATP-bound conformation (Fig. 4a). Using immunoprecipitation without crosslinking, they showed that these closed conformation HSP90 mutants can stably bind to HSF1, and that N-terminal HSP90 inhibitors, like 17-AAG, disrupt the HSP90-HSF1 interaction (Kijima et al. 2018).

There are conflicting reports regarding GB binding to HSP90 (Davenport et al. 2011; Yim et al. 2016), and GBA has to the best of our knowledge not been studied in the context of HSP90. Here, we investigated if GB and GBA also disrupt the interaction between HSF1 and HSP90α and HSP90β closed form mutants. We co-transfected HEK293 cells with WT HSF1-Myc-His and FLAG-HSP90α E47A or FLAG-HSP90β E42A. In accordance with Kijima and coworkers (Kijima et al. 2018), HSP90α E47A forms a stronger interaction with HSF1 than HSP90β E42A as more HSF1 (Myc) was immunoprecipitated with HSP90α E47A than with HSP90β E42A (Fig. 4b). 17-AAG completely disrupted the interaction between HSF1 and HSP90α E47A and HSP90β E42A. We observed that GB also disrupts the interaction between HSF1 and HSP90, both α and β, albeit with lower efficiency than 17-AAG (Fig. 4b).

In addition to its extremely large repertoire of clients, including multiple oncogenic kinases and key transcription factors, HSP90 also interacts with other components of the protein folding machinery, such as HSP70. As seen in Fig. 4b, endogenous HSP70 was immunoprecipitated with both HSP90α E47A and HSP90β E42A, but this interaction was not affected by 17-AAG or GB. Interestingly, we also observed that endogenous HSF2 was immunoprecipitated by FLAG-tagged HSP90α and β (Fig. 4b). To our knowledge, this is the first report demonstrating an HSP90-HSF2 interaction. Both 17-AAG and GB disrupted the interaction between HSF2 and HSP90α or β but to a different degree than HSF1 (Fig. 4b), suggesting that HSF1 and HSF2 can bind independently to HSP90.

The interaction between HSP90α E47A and HSF1 was more robust, and hence we explored if other compounds known to induce a HSR would disrupt the interaction between HSP90α E47A and HSF1. 17-AAG, as well as HS and celastrol, completely disrupted the interaction between HSF1 and HSP90α (Fig. 4c). GB and GBA also disrupted the interaction, with slightly lower efficacy. HSF2 interaction with HSP90 was disrupted by all treatments, with 17-AAG treatment causing the most effective disruption. Endogenous HSP70 interaction with HSP90α E47A was not disrupted by these different compounds.

Taken together, we show that GB and GBA treatment can disrupt the interaction between HSP90 and HSF1. We also show for the first time that endogenous HSF2 can bind HSP90, and this interaction is disturbed by GB and GBA treatment. We propose that this may be part of the mechanism by which GB and GBA activate the HSR.

## Discussion

Targeting HSP90 has been considered beneficial in cancer treatment with HSP90 having many so-called client proteins that are required for a rapidly growing cell. Unfortunately, most HSP90 inhibitors tested today induce an HSR, which is cytoprotective during cancer treatments (Neckers and Workman 2012). Gambogic acid (GB), a bioactive natural product and potential HSP90-inhibitor, has been shown to kill cancer cells more readily than normal cells, which would support GB as an anticancer drug. However, the molecular mechanisms of GB are still not clear. Here, we show that acute treatments with either GB or its structural analog GBA induce a thiol-dependent HSR in multiple cell lines derived from different cellular origins. We demonstrate that GB and GBA treatment induces an HSR, at least partially by disrupting the HSP90-HSF1 and/or HSP90-HSF2 interaction.

Importantly, our study provides the first demonstration that GBA induces a HSR and is thiol-responsive. Molecules containing α,β-unsaturated ketone moieties are highly reactive and covalently modify a plethora of functional cysteine residues in many proteins (Weerapana et al. 2008). Thiol-reactive compounds have previously been shown to activate HSF1 in yeast and to induce a HSR in mammalian cells (Dayalan Naidu and Dinkova-Kostova 2017; Santagata et al. 2012; Trott et al. 2008; Wang et al. 2012). GB and GBA contain an α,β-unsaturated ketone moiety, and reducing this moiety at carbons C9-C10 renders GB inactive (Han et al. 2005). We show that the HSR, which is induced by GB or GBA, is inhibited in the case GB, or GBA is incubated with excess free thiols (Fig. 2). Our observations are corroborated by previous studies showing that GB can bind covalently to cysteine residues of different proteins, a process that is inhibited by excess free thiol such as DTT and thiol-containing antioxidants (Palempalli et al. 2009; Seo et al. 2019; Yang et al. 2012). GB has been shown to enhance the cytotoxic effects of certain chemotherapeutic agents in different cancer cells (Banik et al. 2018). However, it will be challenging to use GB and GBA as specific cancer drugs due to the thiol-reactive nature of GB and GBA, as they may interact with many proteins in the cell.

Second, we bring novelty by showing that HSF2 participates in a complex with HSP90 and in the regulation of HSR by GB and GBA. We show that GB- and GBA-induced HSR is HSF1-dependent and modulated by HSF2. Previous studies have showed that HSF2 also modulates the HS-induced HSR (Joutsen et al. 2020; Östling et al. 2007; Vihervaara et al. 2013) and in response to various stimuli (El Fatimy et al. 2014; Sandqvist et al. 2009). Both HSF1 and HSF2 are implicated in cancer, and HSF2 is downregulated in malignant cancers (Björk et al. 2016; Dai 2018; Puustinen and Sistonen 2020). HSF2 functions as a suppressor of prostate cancer invasion (Björk et al. 2016). To our knowledge, the role of HSF2 in response to HSP90 inhibitors has not previously been thoroughly studied. Interestingly, a recent study showed that *HSF2* KO cells are more sensitive to HSP90 inhibitors; therefore, further studies regarding the role of HSF2 in the context of HSP90 inhibition is of importance (Joutsen et al. 2020).

Finally, we also bring new insights to the mechanisms of action of GB and GBA. Here we show that both GB and GBA treatments can disrupt the interaction between HSP90 and HSF1 and/or HSP90 and HSF2, suggesting that GB and GBA are mechanistically similar to N-terminal inhibitors. N-terminal, but not C-terminal, HSP90 inhibitors disrupt the interaction between HSF1 and closed form HSP90 mutants (Kijima et al. 2018). Davenport and coworkers reported, using surface plasmon resonance, that GB binds to the N-terminal part of HSP90, whereas Yim and coworkers reported that biotinylated GB binds to the middle domain of HSP90 (Davenport et al. 2011; Yim et al. 2016). Kijima and coworkers mapped the interaction between closed form HSP90 mutants and HSF1 and determined that both HSF1 heptad repeats HR-A/B and a part of the regulatory RD domain are required for interaction with HSP90 (Kijima et al. 2018). HSF2 also contains heptad repeats required for trimerization with HSF1, whereas the regulatory domain of HSF2 markedly differs from that of HSF1 (Gomez-Pastor et al. 2018). From our results, we cannot determine if HSF2 directly binds to HSP90 as a potential homotrimer or if the interaction is via its trimerization with HSF1. Importantly, our study points out the need of including HSF2 in further studies regarding the interactions between HSP90 and HSF1. HSP90 has long been assumed to participate in keeping HSF1 monomers from trimerizing in the cytosol but has recently been shown to bind to HSF1 trimers and remove them from HSEs (Kijima et al. 2018). HSP90β also takes part in the activation of HSF1 by lowering the temperature required for heat-induced HSF1 trimerization (Hentze et al. 2016). It is likely that HSP90 participates in the regulation of HSR in a more prevalent and complex manner than previously anticipated, to which HSF2 adds an additional exciting and novel aspect, with potential aspects of cancer treatment.

In conclusion, we show that the natural products GB and GBA, isolated from gamboge resin, induce a strong HSR, in a thiol-dependent manner, and lead to the release of HSF1 and HSF2 from HSP90. The GB and GBA-dependent activation of the HSF-pathway should be taken into account when using these compounds in different studies. In particular, this is a limiting step in using them in anticancer therapies; however, there may be other pathologies, where an activation of the HSR by GB or GBA would be beneficial, through, for example, neuroprotective effects.
